# Prevalence of sexually transmitted infections with *Chlamydia trachomatis, Neisseria gonorrhoeae, Mycoplasma genitalium* and *Trichomonas vaginalis*: findings from the National Survey of Sexual Lifestyles, Attitudes and Health, Slovenia, 2016 to 2017

**DOI:** 10.2807/1560-7917.ES.2022.27.14.2100284

**Published:** 2022-04-07

**Authors:** Irena Klavs, Maja Milavec, Lina Berlot, Tanja Kustec, Marta Grgič-Vitek, Darja Lavtar, Metka Zaletel, Andrej Golle, Darja Duh, Tjaša Žohar Čretnik

**Affiliations:** 1 National Institute of Public Health, Ljubljana, Slovenia; 2 National Laboratory of Health, Environment and Food, Maribor, Slovenia

**Keywords:** *Chlamydia trachomatis*, *Neisseria gonorrhoeae*, *Mycoplasma genitalium*, *Trichomonas vaginalis*, survey, general population, prevalence, Slovenia

## Abstract

**Background:**

To inform prevention and control of sexually transmitted infections (STIs), we need reliable prevalence estimates.

**Aim:**

One objective of the Slovenian National Survey of Sexual Lifestyles, Attitudes and Health was to estimate the prevalence of STIs with *Chlamydia trachomatis*, *Neisseria gonorrhoeae*, *Mycoplasma genitalium* and *Trichomonas vaginalis*.

**Methods:**

Data were collected between October 2016 and July 2017 in a probability sample of the general population aged 18–49 years. Computer-assisted face-to-face interviewing and self-completion of questionnaires were used. Respondents were invited to provide urine samples to be tested for STIs.

**Results:**

Of 1,929 survey participants, 1,087 individuals provided urine samples which were tested confidentially for *C. trachomatis* and a subset (n = 1,023) were tested anonymously for the other STIs. The prevalence of *C. trachomatis* was 0.5% (95% confidence interval (CI): 0.1–1.8) in men and 1.7% (95% CI: 0.9–3.2) in women. Age-specific prevalence was the highest among individuals aged 18–24 years, 2.8% (95% CI: 0.7–10.6) in men and 4.7% (95% CI: 1.7–12.3) in women. *N. gonorrhoea* was not detected. Prevalence of *M. genitalium* was 0.5% (95% CI: 0.1–2.2) in men and 0.3% (95% CI: 0.1–1.1) in women; the highest prevalence was among men aged 25–34 years (1.1%; 95% CI: 0.2–7.5) and women aged 35–49 years (0.5%; 95% CI: 0.1–2.0). *T. vaginalis* was detected in the sample from one woman (0.2%; 95% CI: 0.1–1.2).

**Conclusion:**

The substantial prevalence of *C. trachomatis* among young adults suggests gaps in testing, diagnosis and treatment.

## Introduction

Sexually transmitted infections (STIs) are among the most common communicable conditions and affect people worldwide. In 2016, the sixty-ninth World Health Assembly adopted the Global health sector strategy on sexually transmitted infections, 2016–2021 [1]. In 2019, the World Health Organization (WHO) published estimates for 2016 of the global prevalence and incidence of the four most common curable STIs in men and women aged 15–49 years [2]. Based on a systematic review of articles published between 1 January 2009 and 29 July 2018, prevalence estimates for three of these STIs for the WHO European Region were: (i) *Chlamydia trachomatis* 2.2% (95% confidence interval (CI): 1.5–3.0) for men and 3.2% (95% CI: 2.5–4.2) for women; (ii) *Neisseria gonorrhoeae* 0.3% (95% CI: 0.1–0.5) for men and 0.3% (95% CI: 0.1–0.6) for women and; (iii) *Trichomonas vaginalis* 0.2% (95% CI: 0.1–0.3) for men and 1.6% (95% CI: 1.1–2.3) for women [2]. These prevalence estimates for the European Region were based on studies of different population groups of which only one, the third British National Survey of Sexual Attitudes and Lifestyles (Natsal-3), was performed in a national probability sample of the general population aged 16–44 years [3,4]. The need to expand data collection efforts at country level and provide an initial baseline to monitor the progress of the WHO health sector strategy on STIs was emphasised [2].

In Slovenia, surveillance of selected STIs, including infections with *C. trachomatis* and *N. gonorrhoeae*, has been based on universal mandatory reporting of newly diagnosed cases. This, however, does not reflect the true occurrence of STIs in the population because STIs are often asymptomatic, undiagnosed and also under-reported [5]. Between 2014 and 2018, reported annual rates of newly diagnosed cases of chlamydia infection in Slovenia were ca 10 times lower than the corresponding overall annual rates reported for European Union/European Economic Area (EU/EEA) countries [6]. These low rates reported for Slovenia were mostly because of low annual diagnostic testing rates, which during the 2014–18 period varied between 175 and 220 tests per 100,000 population, and in part because of under-reporting [5,7]. In contrast, a high prevalence of chlamydia infection (4.7%; 95% CI: 2.2–8.5) among sexually experienced men and women aged 18–24 years was estimated in the first Slovenian National Survey of Sexual Lifestyles, Attitudes and Health in 1999–2000 [8].

Reported rates for gonorrhoea during 2014–18 in Slovenia were more similar to overall annual rates reported from EU/EEA countries [5,9]. Infections with *Mycoplasma genitalium* and protozoan *T. vaginalis* have not been reportable in Slovenia. By 2016, reliable prevalence estimates for the latter three STIs in the general population of Slovenia were not available.

To inform STI prevention and control policies in Slovenia and to complement routinely available STI surveillance data, one of the objectives of the second Slovenian National Survey of Sexual Lifestyles, Attitudes and Health was to estimate the prevalence of *C. trachomatis*, *N. gonorrheae*, *M. genitalium* and *T. vaginalis* in the general population aged 18–49 years.

## Methods

### Participants and procedures

A stratified two-stage probability sample of the general population aged 18–49 years consisting of 4,000 individuals was selected from the central population registry by the Statistical Office of Slovenia according to their standard procedures. Within the 12 statistical regions of Slovenia (explicit strata), we defined implicit strata according to six sizes and types of communities: (i) rural communities with fewer than 2,000 inhabitants; (ii) non-rural communities with fewer than 2,000 inhabitants; (iii) communities with 2,000–9,999 inhabitants; (iv) those with 10,000–100,000 inhabitants and two cities with more than 100,000 inhabitants: (v) Ljubljana and (vi) Maribor.

The whole sampling frame included primary sampling units, which were either enumeration areas on their own or several smaller ones joined together. Primary sampling units were sorted by size and type of community within each statistical region. Subsequently a systematic sample of 500 primary sampling units was selected with replacement and independently from all 12 statistical regions. Each primary sampling unit was selected with a probability proportional to the size of the eligible population. Then, eight secondary sampling units, individuals aged 18-49 years, were selected by systematic random procedure within each primary sampling unit.

Interviews were performed at respondents’ homes between October 2016 and July 2017 using a combination of computer-assisted face-to-face interviewing and self-completion of questionnaires that included more sensitive questions.

After the interview, we invited all respondents to provide a first-void urine (FVU) sample for confidential testing for *C. trachomatis*. Respondents were told that they would be notified and referred to treatment if chlamydia infection was diagnosed. In addition, we asked respondents to consent to delayed anonymous testing of the FVU sample for other STIs, when resources for such testing became available. We explained to participants that such delayed testing for other STIs would be performed for research purposes to obtain general population prevalence estimates for selected STIs, and that participants would not receive the results. Once written informed consent for voluntary confidential testing for *C. trachomatis* and, in most cases, separate informed consent for delayed anonymous testing for other STIs was obtained, we instructed participants on how to provide a FVU sample using the FirstBurst (Diagnostics for the Real World, Cambridge, United Kingdom) device. This device collects the first 4–5 mL of voided urine, thus yielding a higher load of *C. trachomatis* than the regular urine cup [10]. Interviewers posted FVU samples to the laboratory within 24 h.

### Laboratory methods and notification of infected individuals

We detected *C. trachomatis* with a specific in-house real-time polymerase chain reaction (PCR) test targeting both cryptic plasmid and bacterial chromosome [11]. Positive results were confirmed by Sanger sequencing of the amplicon. We notified individuals with diagnosed chlamydia infection, referring men to their general practitioner (GP) and women to their GP or gynaecologist for treatment.

After testing for *C. trachomatis*, only FVU samples of individuals who consented to delayed anonymous testing for other STIs were stored for further testing. Each sample was marked with a unique identifier that enabled us to link the anonymous test result for other STIs with the information collected from the individual during the interview. We tested for *N. gonorrhoeae*, *M. genitalium* and *T. vaginalis* with a commercially available multiplex real-time PCR, FTD Urethritis plus kit (Fast-track Diagnostics, Luxembourg). To avoid false-negative results, the human housekeeping gene was amplified in all tested samples. The test also detected *C. trachomatis*. The number of *C. trachomatis* positive results was equal to the number of positive results obtained with the in-house real-time PCR of all those individuals who consented to delayed anonymous testing for other STIs. The age and sex characteristics of individuals who tested positive for *C. trachomatis* matched across both tests.

### Statistical analysis

Statistical analyses were done in Stata version 15 (StataCorp, College Station, Texas, United States). Survey response and FVU sample collection rates for confidential testing for infection with *C. trachomatis* and for anonymous testing for other STIs were calculated from unweighted data. We computed two sets of weights. The first set was to adjust for differences between confidentially tested FVU samples for *C. trachomatis* infection and population estimates for Slovenia according to sex, age (18–19, 20–24, 25–29, 30–34, 35–39, 40–44, 45–49 years), region (Pomurska, Podravska, Koroška, Savinjska, Zasavska, Posavska, Jugovzhodna Slovenija, Osrednjeslovenska, Gorenjska, Primorsko-notranjska, Goriška, Obalno-kraška), and size and type of community (< 2,000 rural; < 2,000 non-rural; 2,000–9,999; 10,000–100,000; > 100,000 Maribor; > 100,000 Ljubljana). The second set was to adjust for differences between anonymously tested FVU samples for three other STIs and the population estimates for Slovenia according to sex, age, region, and size and type of community. We used iterative proportional fitting method for consecutive adjusting of weights for each individual so that our weighted samples (survey, FVU sample tested for *C. trachomatis* and FVU sample tested for other STIs) closely matched the distribution of the Slovenian population aged 18–49 years according to these demographic characteristics in the central population registry on 1 January 2017. The sum of both sets of weights equalled the number of tested FVU samples (average weight was 1). For the first set of weights, the minimum weight was 0.42 and the maximum 2.14. For the second set of weights, the minimum weight was 0.39 and the maximum 2.30.

In all other analyses we accounted for stratification, clustering and weighting of the sample using Stata svy commands. We present weighted prevalence estimates together with 95% CI for *C. trachomatis, N. gonorrheae, M. genitalium and T. vaginalis* infections in men and in women of different ages and overall. When the point prevalence estimates were 0.0% in any group of individuals, we presented them together with one-sided binomial 97.5% CI (exact method). Since a substantial proportion of the population aged 18–24 years was not sexually experienced, we also present corresponding prevalence estimates for *C. trachomatis* infections in sexually experienced men and women aged 18–24 years. We defined sexually experienced individuals as those who had ever had heterosexual penetrative intercourse (vaginal, oral or anal) and/or homosexual penetrative intercourse (oral or anal) or genital contact.

## Results

**Figure 1:**
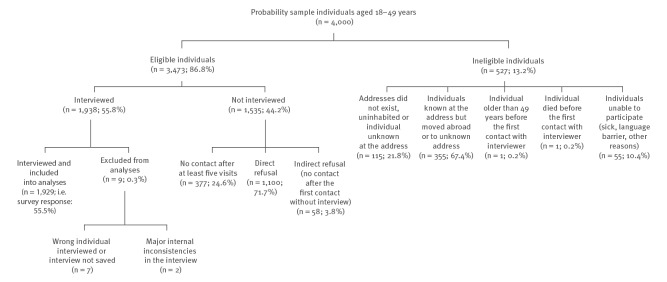
Response in the National Survey of Sexual Lifestyles, Attitudes and Health, general population aged 18–49 years, Slovenia, October 2016–July 2017 (n = 1,929)

Survey response is described in detail in Figure 1. Of 3,473 eligible individuals, 1,929 were interviewed and included in the analyses, corresponding to a survey response rate of 55.5%.

**Figure 2:**
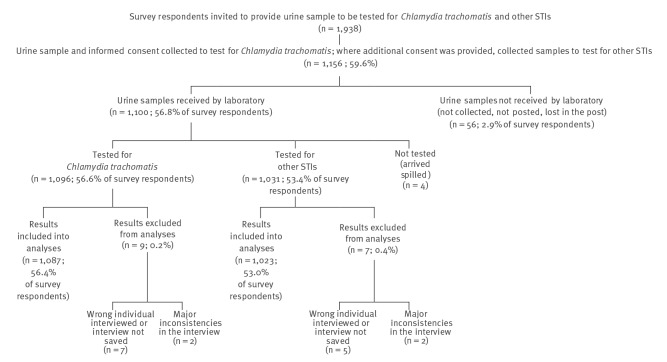
Urine sample collection and testing rates for *Chlamydia trachomatis, Neisseria gonorrheae, Mycoplasma genitalium* and *Trichomonas vaginalis,* general population aged 18–49 years, Slovenia, October 2016–July 2017 (n = 1,087)

Urine sample collection and testing rates for *C. trachomatis* and for *N. gonorrheae*, *M. genitalium* and *T. vaginalis* are described in detail in Figure 2.

STIs: sexually transmitted infections.

The results of 1,087 FVU samples tested for *C. trachomatis* were included in the analyses, corresponding to 56.4% of survey respondents (1,087/1,929) or 31.3% of all eligible individuals (1,087/3,473). As not all individuals who consented to confidential testing for *C. trachomatis* consented to delayed anonymous testing for other STIs, 1,023 FVU samples were tested for the other three STIs and respective results included into analyses, corresponding to 53.0% of survey respondents or 29.5% of all eligible individuals.

Table 1 shows survey response rates, confidential urine sample testing rates for *C. trachomatis* and anonymous testing rates for three other STIs separately for men and women according to their age group, community size and type and region.

**Table 1:** Survey response rates and rates of urine sample testing for sexually transmitted infections according to sex, age group, community size and type and statistical region, Slovenia, October 2016–July 2017 (n = 3,473)

Categories	Men							Women						
	Number of eligible individuals	Interviews conducted		Urine samples tested				Number of eligible individuals	Interviews conducted		Urine samples tested			
				*Chlamydia trachomatis*		Three STIs^a^					*Chlamydia trachomatis*		Three STIs^a^	
		n	% eligible	n	% eligible	n	% eligible		n	% eligible	n	% eligible	n	% eligible
Age (years)														
18–19	69	40	58.0	21	30.4	20	29.0	66	46	69.7	25	37.9	24	36.4
20–24	189	114	60.3	59	31.2	56	29.6	211	141	66.8	87	41.2	83	39.3
25–29	260	138	53.1	65	25.0	63	24.2	204	120	58.8	71	34.8	68	33.3
30–34	267	148	55.4	79	29.6	76	28.5	250	153	61.2	96	38.4	91	36.4
35–39	342	160	46.8	83	24.3	79	23.1	338	198	58.6	125	37.0	116	34.3
40–44	308	147	47.7	70	22.7	65	21.1	353	192	54.4	125	35.4	115	32.6
45–49	312	155	49.7	75	24.0	71	22.8	304	177	58.2	106	34.9	96	31.6
Community size														
< 2,000 rural	456	284	62.3	147	32.2	141	30.9	432	288	66.7	191	44.2	180	41.7
< 2,000 non-rural	538	279	51.9	138	25.7	132	24.5	504	312	61.9	185	36.6	171	33.9
2,000–9,999	256	141	55.1	67	26.2	63	24.6	298	176	59.1	112	37.6	106	35.6
10,000–100,000	218	101	46.3	44	20.2	40	18.3	213	118	55.4	63	29.6	57	26.8
> 100,000; Maribor	72	33	45.8	24	33.3	23	31.9	70	35	50.0	25	35.7	24	34.3
> 100,000; Ljubljana	207	64	30.9	32	15.5	31	15.0	209	98	46.9	59	28.2	55	26.3
Statistical region														
Gorenjska	182	81	44.5	33	18.1	31	17.0	190	103	54.2	59	31.1	56	29.5
Goriška	93	46	49.5	22	23.7	20	21.5	103	71	68.9	45	43.7	41	39.8
Jugovzhodna Slovenija	123	64	52.0	34	27.6	34	27.6	123	78	63.4	48	39.0	43	35.0
Koroška	49	33	67.3	10	20.4	10	20.4	63	46	73.0	30	47.6	30	47.6
Obalno-kraška	78	38	48.7	24	30.8	24	30.8	91	50	54.9	25	27.2	25	27.2
Osrednjeslovenska	452	199	44.0	98	21.7	94	20.8	444	216	48.6	118	26.6	112	25.2
Podravska	277	155	56.0	93	33.6	88	31.8	258	172	66.7	113	43.8	105	40.7
Pomurska	99	58	58.6	31	31.3	31	31.3	99	68	68.7	49	49.5	49	49.5
Posavska	72	48	66.7	31	43.1	30	41.7	55	33	60.0	25	45.5	24	43.6
Primorsko-notranjska	51	26	51.0	15	29.4	14	27.5	37	23	62.2	21	56.8	20	54.1
Savinjska	218	129	59.2	49	22.5	42	19.3	215	144	67.0	86	40.0	73	34.0
Zasavska	53	25	47.2	12	22.6	12	22.6	48	23	47.9	16	33.3	15	31.3
Total	1,747	902	51.6	452	25.9	430	24.6	1,726	1,027	59.5	635	36.8	593	34.4

STIs: sexually transmitted infections.

^a^ *Neisseria gonorrhoeae*, *Mycoplasma genitalium* and *Trichomonas vaginalis.*

A total of 16 individuals (unweighted count) were found to be infected with at least one of the four STIs. All were sexually experienced and, except for one woman, all reported more than one partner of the opposite sex during their lifetime. Nine individuals reported five or more partners. Of four men, neither of whom had homosexual history, two were infected with *C. trachomatis* (both aged 18–24 years) and two with *M. genitalium* (one aged 25–34 years and one aged 35–49 years). None reported previous diagnoses of STIs. From 12 women, two of whom in addition to heterosexual also had homosexual history, nine were infected with *C. trachomatis* (four aged 18–24 years, four aged 25–34 years, and one aged 35–49 years), one with *M. genitalium* (aged 35–49 years), one with both *C. trachomatis* and *M. genitalium* (aged 35–49 years), the only coinfection detected, and one with *T. vaginalis* (aged 25–34 years). Among women with chlamydia infection, one reported a previous diagnosis of bacterial vaginosis and one a previous diagnosis of candidiasis.

Table 2 shows weighted prevalence estimates for STIs with *C. trachomatis*, *N. gonorrhoeae*, *M. genitalium* and *T. vaginalis* for individuals aged 18–49 years by sex.

**Table 2:** Prevalence of *Chlamydia trachomatis, Neisseria gonorrhoeae, Mycoplasma genitalium* and *Trichomonas vaginalis* in the general population aged 18–49 years by sex, Slovenia, October 2016–July 2017 (n = 1,087)

Sex	Aged 18–24 years		Aged 25–34 years		Aged 35–49 years		All ages (18–49 years)	
	Proportion		Proportion		Proportion		Proportion	
	%	95% CI	%	95% CI	%	95% CI	%	95% CI
**Men**								
*Chlamydia trachomatis*	2.8	0.7–10.6	0.0	0.0–2.1^a^	0.0	0.0–1.2^a^	0.5	0.1–1.8
Denominator: UWT; WT	80; 91		144; 174		228; 300		452; 565	
*Neisseria gonorrhoeae*	0.0	0.0–4.2^a^	0.0	0.0–2.2^a^	0.0	0.0–1.3^a^	0.0	0.0–0.7^a^
*Mycoplasma genitalium*	0.0	0.0–4.2^a^	1.1	0.2–7.5	0.3	0.0–1.7	0.5	0.1–2.2
*Trichomonas vaginalis*	0.0	0.0–4.2^a^	0.0	0.0–2.2^a^	0.0	0.0–1.3^a^	0.0	0.0–0.7^a^
Denominator: UWT; WT	76; 86		139; 164		215; 282		430; 532	
**Women**								
*Chlamydia trachomatis*	4.7	1.7–12.3	2.2	0.8–6.0	0.4	0.1–1.5	1.7	0.9–3.2
Denominator: UWT; WT	112; 86		167; 160		356; 276		635; 522	
*Neisseria gonorrhoeae*	0.0	0.0–4.5^a^	0.0	0.0–2.4^a^	0.0	0.0–1.4^a^	0.0	0.0–0.7^a^
*Mycoplasma genitalium*	0.0	0.0–4.5^a^	0.0	0.0–2.4^a^	0.5	0.1–2.0	0.3	0.1–1.1
*Trichomonas vaginalis*	0.0	0.0–4.5^a^	0.5	0.1–3.7	0.0	0.0–1.4^a^	0.2	0.1–1.2
Denominator: UWT; WT	107; 81		159; 151		327; 260		593; 491	
**All men and women**								
*Chlamydia trachomatis*	3.7	1.7–8.2	1.1	0.4–3.0	0.2	0.0–0.7	1.0	0.6–1.9
Denominator: UWT; WT	192; 177		311; 334		584; 576		1,087; 1,087	
*Neisseria gonorrhoeae*	0.0	0.0–2.2^a^	0.0	0.0–1.2^a^	0.0	0.0–0.7^a^	0.0	0.0–0.4^a^
*Mycoplasma genitalium*	0.0	0.0–2.2^a^	0.6	0.1–4.0	0.4	0.1–1.2	0.4	0.1–1.1
*Trichomonas vaginalis*	0.0	0.0–2.2^a^	0.3	0.0–1.8	0.0	0.0–0.7^a^	0.1	0.0–0.6
Denominator: UWT; WT	183; 167		298; 315		542; 542		1,023; 1,023	

CI: confidence interval; UWT: unweighted count of individuals; WT: weighted count of individuals.

^a^ One-sided binomial 97.5% CI.

### *Chlamydia trachomatis* infections

The weighted prevalence of infections with *C. trachomatis* among men was 0.5% (95% CI: 0.1–1.8) and 1.7% (95% CI: 0.9–3.2) among women. It was highest among the 18–24 years age group at 2.8% (95% CI: 0.7–10.6) among men and 4.7% (95% CI: 1.7–12.3) among women. Prevalence decreased with older age in both sexes (both p < 0.05). Since almost all respondents at least 25 years old who provided FVU sample were already sexually experienced (98.3% among men; 99.0% among women), the prevalence estimates for chlamydia infection among all men and women, in comparison to those men and women who already had sex, were almost identical for both older age groups (25–34 and 35–49 years old). However, there was a higher proportion of 18–24-year-old individuals who provided a FVU sample and who had not yet had sex (14.8% among men; 15.7% among women). Thus, in comparison to all 18–24-year-old individuals, the point prevalence estimates of chlamydia infections were higher among those who had already had sex: 3.4% (95% CI: 0.9–12.5) among men, 5.6% (95% CI: 2.0–14.4) among women and 4.5% (95% CI: 2.0–9.7) among both.

None of the 12 individuals aged 18–49 years with *C. trachomatis* infection (unweighted count) reported having had it diagnosed previously and half (6/12) reported only one heterosexual partner in the previous year. The two men aged 18–24 years infected with *C. trachomatis* reported that they had never had urethral discharge and that they visited the general practitioner during the month before the interview. Nine of the 10 women with *C. trachomatis* infection reported at least one visit to a general practitioner in the previous year and six women also reported at least one visit to a gynaecologist in the previous year. Of four women aged 18–24 years, three reported at least one visit to a gynaecologist during last year.

### *Neisseria gonorrhoeae, Mycoplasma genitalium* and *Trichomonas vaginalis* infections

We detected no cases of infection with *N. gonorrhoeae*, the estimated weighted prevalence of gonorrhoea in the general population of Slovenia aged 18–49 years was low (0.0%, one-sided binomial 97.5% CI: 0.0–0.4).

Weighted prevalence of infections with *M. genitalium* infections was 0.5% (95% CI: 0.1–2.2) among all men aged 18–49 years and 0.3% (95% CI: 0.1–1.1) among women aged 18–49 years. No cases were detected in the youngest age group of men and women (18–24 years old) and among women aged 25–34 years. None of the two men (unweighted count) with *M. genitalium* infections reported having had such a diagnosis previously, however, one of them reported urethral discharge during the previous month and that he had visited a general practitioner in the previous month. Also, neither of the two women with *M. genitalium* infection reported having previously received such a diagnosis and both reported having visited either a general practitioner or a gynaecologist during the previous year.

*T. vaginalis* infection was detected in one woman aged 25–34 years. Corresponding estimated weighted prevalence among all women was 0.2% (95% CI: 0.1–1.2) and 0.0% (one-sided binomial 97.5% CI: 0.0–0.7) among all men. The woman reported not having had *T. vaginalis* infection diagnosed previously. She had visited the GP during the previous year but not in the previous month, and had visited the gynaecologist during last 5 years, but not within the previous year.

## Discussion

To inform STIs prevention and control policies in Slovenia, we have obtained prevalence estimates of *C. trachomatis*, *N. gonorrhoea*, *M. genitalium* and *T. vaginalis* infections in the general population aged 18–49 years for October 2016–July 2017. Although the need to expand STIs data collection efforts at country level and provide an initial baseline to monitor the progress of the WHO health sector strategy on STIs was emphasised [2], to date in Europe, reliable prevalence estimates for these four STIs from a national survey of the general population using a probability sample with a wide age range have only been published for Britain [3,4,12].

Although reported rates of new diagnoses of chlamydia infection in Slovenia have remained low [5], our results for 2016–17, ca 15 years after the first prevalence estimates for infections with *C. trachomatis* among the general population aged 18–49 years were available, show that prevalence among sexually experienced 18–24-year-old individuals has remained substantial, especially among women (5.6%; 95% CI: 2.0–14.4). This implies that a large proportion of infections with *C. trachomatis* still remains undiagnosed and untreated. Our results provide support for considering the introduction of annual opportunistic testing for *C. trachomatis* for sexually active women younger than 25 years who attend sexual health services, to detect and treat infections early and prevent long-term complications for reproductive health. This is in line with the 2015 European guideline on the management of *C. trachomatis* infections, which also recommends testing of all men younger than 25 years of age, who attend STIs and sexual health clinics [13]. The implementation of opportunistic testing of sexually active women younger than 25 years of age in primary healthcare outpatient gynaecology services that perform sexual and reproductive health services for women in Slovenia should be considered. Good coverage of this target population is guaranteed, as a great majority of sexually experienced women aged 18–24 years reported to have visited a gynaecologist in the previous year (77.7%; 95% CI: 68.9–84.5). Guidelines in many high-income countries recommend *C. trachomatis* screening for all sexually active young (< 25 years of age) women [14-17]. In addition, sexual and reproductive health education, promotion of condom use, appropriate case management and partner notification should be part of a Slovenian national chlamydia infection control strategy, together with systems for monitoring and evaluation [18,19].

Since we did not find a single infection with *N. gonorrhoeae*, we can conclude that in 2016–17, the prevalence of *N. gonorrhoeae* infection was relatively low in the general population of Slovenia aged 18–49 years, however, we may have underestimated its prevalence among men because of participation bias of men who have sex with men (MSM). Reported rates of new diagnoses of infection with *N. gonorrhoeae* show that MSM are disproportionally affected [5]. In 2018, 90% of new diagnoses of gonorrhoea were reported among men and, among those, 54% were among MSM [5]. Men who have sex with men may have been underrepresented in our survey sample. While the European MSM Internet survey performed in 2009 estimated that the size of the population of MSM in Slovenia exceeded 3% of the adult male population [20], only 1.2% (95% CI: 0.6–2.2) of the male respondents in our study reported having had sex with men during the previous 5 years.

Our first reliable prevalence estimate for infections with *M. genitalium* in the general population aged 18–49 years in 2016–17 provided evidence that this STI was also relatively common. However, in contrast to chlamydia infection, the prevalence was higher among older individuals that are not the primary targets of STIs prevention interventions. Higher prevalence of *M. genitalium* infection in older age groups was also observed in the Natsal-3 study [12]. The European guideline on *M. genitalium* infections has been published in 2016 [21]. The public health value of testing asymptomatic persons for *M. genitalium* has not been established [21].

Our first general population probability sample prevalence estimates of *T. vaginalis* infections indicated that it was also rare in the general population of Slovenia aged 18–49 years in 2016–17.

We used methods similar to the Natsal-3 performed in 2010–12 and the age ranges of the general population examined were only slightly different, hence our prevalence estimates for these four STIs are best comparable to the published British estimates [3,4,12]. It is worth noting that both Slovenian and British point prevalence estimates for three of these STIs, *C. trachomatis*, *N. gonorrhoeae*, and *T. vaginalis*, were lower than recently published respective WHO point prevalence estimates for the general population of the European Region aged 15–49 years in 2016 [2]. Although prevalence of these STIs may differ between European countries, our results and the British results suggest that WHO estimates for the European Region might have been overestimated. They were based on the results of a limited number of studies from a few countries (21 studies estimating chlamydia prevalence from 10 EU countries and one from Georgia; eight studies estimating gonorrhoea prevalence from five EU countries; and four studies estimating trichomoniasis prevalence from three EU countries) [2]. Notably, these studies were performed in different population groups (e.g., attendees of antenatal care clinic, urology and gynaecology clinics, outpatient clinic, cervical cancer screening programmes, young women vaccinated against HPV, sexually active students, etc.) with often narrow age ranges, while only the included Natsal-3 study was performed in a national probability sample of the general population with a wide age range [2]. In addition to limited STIs prevalence data available, the authors listed several other limitations including different age groups of people studied and different diagnostic tests used [2]. Another limitation mentioned by the authors was that general population samples may underestimate the prevalence of those STIs that disproportionally affect key populations, which could result in an underestimation of prevalence in a probability sample of the general population, if members of key populations are less likely to participate [2]. For example, gonorrhoea is known to disproportionally affect MSM in EU/EEA countries. In 2018, MSM accounted for 48% and heterosexual men and women for 43% of all reported cases in EU/EEA countries (for 9% transmission group was reported as unknown) [9]. In view of this, respective WHO point prevalence estimates for gonorrhoea among men and women aged 15–49 years in 2016 in the European Region (men: 0.3%; 95% CI: 0.1–0.5; women: 0.3%; 95% CI: 0.1–0.6) seem inconsistent [2].

The European Centre for Disease Prevention and Control (ECDC) coordinated the surveillance of selected STIs in the EU/EEA, including chlamydia infection and gonorrhoea but not infections with *M. genitalium* and *T. vaginalis*, and this has been based on reporting of newly diagnosed cases from EU/EEA countries [6,9]. In 2018, national annual reported rates in EU/EEA countries varied between 0 and 578 cases per 100,000 population for chlamydia infection and between 0.3 and 93.2 cases per 100,000 population for gonorrhoea [6,9]. ECDC concluded that differences in notification rates across European countries probably reflect differences in testing policies, access to testing services as well as differences in surveillance systems between countries, rather than just actual differences [6,9]. For example, the annual reported rate for newly diagnosed cases of chlamydia infection in Slovenia in 2018 was 16.1 per 100,000 population, while it was 365.7 per 100,000 population in the United Kingdom (UK) [5,6]. However, the annual diagnostic testing rate for *C. trachomatis* in Slovenia for 2018 was 220 tests per 100,000 population, which was lower than reported chlamydia infection notification rate for the UK [5,6]. In contrast, general population prevalence estimates of chlamydia infections in Slovenia in 2016–17 and Great Britain in 1999–2000 were quite similar [3]. It is well known that more testing brings more diagnoses. Thus, without information about respective diagnostic testing rates, comparing reported rates of new diagnoses between EU/EEA countries may lead to distorted conclusions. To better interpret annual reported rates of chlamydia infection in EU/EEA countries, available information about overall national annual diagnostic testing rates should be collected and presented together with reported rates, as is done for HIV surveillance [22]. Reported new STI diagnoses data should also be regularly triangulated with available prevalence data for the general population or conveniently accessible sentinel populations, for example women in perinatal care or convenience samples of young adults eligible for screening [2,23]. In 2014, ECDC published a systematic review of literature about the prevalence of *C. trachomatis* infection in general population groups of different ages for several EU/EEA countries [19]. Finally, ECDC recently published the results of a study on the burden of communicable diseases in Europe, where chlamydia infection ranked tenth among 31 examined communicable diseases, with an estimated 4.6 (95% CI: 2.2–9.0) disability adjusted life years per 100,000 population annually in EU/EEA countries during 2009–13 [24]. Such a composite health measure supports informed policy formulation based on the relative importance of different communicable diseases [24].

Methodological strengths of our survey included a reliable general population sampling frame, the central population registry, which is not available in all countries, and use of methods adapted from the Natsal-1 that were pre-tested and piloted in Slovenia during preparations for the first Slovenian National Survey of Sexual Lifestyles, Attitudes and Health [8,25-27]. The survey response rate of 55.5% was acceptable, as was the FVU sample collection rate (56.4%), both of which were similar to the survey response (57.7%) and FVU sample collection (60.0%) rates in the Natsal-3 [3]. To minimise non-participation bias, we weighted our survey data for differential non-response so that all our weighted samples (survey, FVU samples tested for *C. trachomatis* and FVU samples tested for other STIs) closely matched the Slovenian population aged 18–49 years in the central population registry with respect to distribution of characteristics such as sex, age, region, and size and type of community. To minimise reporting bias i.e., selective revealing or suppression of information by study participants, we used computer-assisted self-interviewing for more sensitive questions.

We were able to perform immediate voluntary confidential testing for *C. trachomatis* and notification of participants in case of positive results, similar to our approach in the first Slovenian National Survey of Sexual Lifestyles, Attitudes and Health in 1999–2000 [8,26]. Anonymous testing for the three other STIs was performed later only on FVU samples collected from respondents who signed a separate informed consent form permitting such testing for research purposes only and who were aware that the results would not be returned. The reason for such different approaches to testing for different STIs was a lack of resources during the data collection period and limits of testing accuracy for diagnostic purposes of some STIs in survey conditions and logistical constrains. In the Natsal-3, exclusively voluntary anonymous testing for several STIs, including chlamydia infection, without returning the results with specific consent of participants was performed [3,4,12]. Such approach was developed in consultation with stakeholders, carefully considered for its ethics, piloted and implemented after approval of a research ethics committee [28].

One of the limitations of our survey was a small sample size. Thus, our STI prevalence estimates were less precise than desired. Survey participants who provided FVU samples may not have been fully representative of the general population aged 18–49 years. We may have underestimated the prevalence of gonorrhoea among men since MSM, who tend to be stigmatised, may have been less likely to participate in our survey than other men. In fact, among 430 male respondents, whose FVU samples were tested for *N. gonorrhoeae*, only three men (unweighted count) reported sex with other men in the past year, corresponding to 0.7% of these respondents, which suggests that some participation bias may have occurred.

### Conclusions

Prevalence of infection with *C. trachomatis* was substantial, especially among sexually experienced women aged 18–24 years. Introduction of annual opportunistic testing for *C. trachomatis* for sexually active women younger than 25 years of age who attend sexual health services, to detect and treat infections early and prevent long-term complications for reproductive health should be considered.

Infection with *M. genitalium* was also relatively common, but in contrast to infection with *C. trachomatis*, the prevalence was higher among older individuals that are not the main age group targeted with STI prevention interventions. The prevalence of infections with *N*. *gonorrhoeae* and *T. vaginalis* was low.

Point prevalence estimates for *C. trachomatis*, *N. gonorrhoeae*, and *T. vaginalis* were lower than recently published respective WHO point prevalence estimates for the general population of the European Region, 15–49 years old.

Except for infections with *C. trachomatis*, these are the first such prevalence estimates for a central European country. Monitoring STI prevalence change in the general population with methods providing accurate prevalence estimates is essential to guide prevention and control policies and strategies on national, regional and global level.
